# Factors influencing reversion from virus infection in sweetpotato

**DOI:** 10.1111/aab.12551

**Published:** 2019-11-06

**Authors:** Alexander Ssamula, Anthony Okiror, Liat Avrahami-Moyal, Yehudit Tam, Victor Gaba, Richard W. Gibson, Amit Gal-On, Settumba B. Mukasa, Peter Wasswa

**Affiliations:** 1 Department of Agricultural Production, Makerere University, Kampala, Uganda; 2 Department of Plant Pathology and Weed Research, Agricultural Research Organization–The Volcani Center, Rishon LeZion, Israel; 3 Natural Resources Institute, University of Greenwich, Kent, UK

**Keywords:** East African cultivars, sweetpotato, sweetpotato viruses, USA cultivars, virus reversion

## Abstract

Viruses limit sweetpotato (*Ipomoea batatas*) production worldwide. Many sweetpotato landraces in East Africa are, however, largely virus-free. Moreover, some plants infected by the prevalent Sweet *potato feathery mottle virus* (SPFMV) may be able to revert to virus-free status. In this study, we analysed reversion from SPFMV, *Sweet potato virus C, Sweet potato mild mottle virus, Sweet potato chlorotic stunt virus* (SPCSV) and *Sweet potato leaf curl Uganda virus* using the indicator plant *I. setosa* and PCR/reverse-transcriptase PCR. We also investigated environmental factors (temperature and soil nutrients) that may influence reversion from virus infection. We tested reversion in the East African cultivars New Kawogo, NASPOT 1 and NASPOT 11, and the United States cultivars Resisto and Beauregard. Reverted plants were asymptomatic and virus was undetectable in assayed parts of the plant. After graft inoculation, only the East African cultivars mostly reverted at a high rate and from most viruses though cultivar Beauregard fully reverted following sap inoculation with *Sweet potato virus C.* None of the tested cultivars fully reverted from single or double infections involving SPCSV, and reversion was only observed in co-infections involving potyviruses. Root sprouts derived from SPFMV-reverted plants were also virus free. Reversion generally increased with increasing temperature and by improved soil nutrition. Overall, these results indicate variation in reversion by cultivar and that the natural ability of sweetpotato plants to revert from viruses is malleable, which has implications for both breeding and virus control.

## 1 INTRODUCTION

Sweetpotato (*Ipomoea batatas* (L.) Lam.) is an important root crop, with world production of more than 100 million tonnes annually. In East Africa, sweetpotato is second only to cassava in importance as a starch staple (Food and Agriculture Organization, 2016). Sweetpotato is both a staple and a food security crop in most of sub-Saharan Africa and is grown mainly by smallholder farmers (Andrade et al., 2009; Bashasha, Mwanga, Ocitti p’Obwoya, & Ewell, 1995).

Many sweetpotato-infecting viruses have been reported in Uganda. These include *Sweet potato feathery mottle virus* (SPFMV; family *Potyviridae*, genus *Potyvirus*), *Sweet potato chlorotic stunt virus* (SPCSV; family *Closteroviridae*, genus *Crinivirus*), *Sweet potato mild mottle virus* (SPMMV; family *Potyviridae*, genus *Ipomovirus*), *Sweet potato chlorotic fleck virus* (SPCFV, family *Flexiviridae*, genus *Carlavirus*), *Sweet potato caulimo-like virus* (SPCLV, family *Caulimoviridae*, genus *Caulimovirus*) and *Sweet potato leaf curl Uganda virus* (SPLCUV; family *Geminiviridae*, genus *Begomovirus*) (Aritua et al., 2007; Mukasa, Rubaihayo, & Valkonen, 2003; Wasswa et al., 2011). The East African strain of SPFMV is widely distributed (Karyeija, Gibson, & Valkonen, 1998; Tairo et al., 2005) and in co-infection with SPCSV, leads to the devastating sweet potato virus disease (SPVD) (Gibson et al., 1998). Other potyviruses have been reported in sweetpotato, for example, *Sweet potato virus C* (SPVC) in Israel (Prakash, Tam, Zeidan, Abu-Ras, & Gaba, 2013).

Sweetpotato is propagated vegetatively using vines as planting material. Farmers often reuse vines from their own fields or neighbouring farmers’ fields (Rachkara, Phillips, Kalule, & Gibson, 2017), a practice that can maintain and spread viruses leading to cultivar degeneration (Adikini, Mukasa, Mwanga, & Gibson, 2015). Most single viral infections cause yield losses, but only minor or transient symptoms (Karyeija et al., 1998), making it difficult to identify infected plants. Unchecked, natural spread occurs under field conditions, and an entire crop can quickly become infected.

Previous surveys have reported at least some virus-free sweetpotato plants on East African farms (Aritua et al., 2007; Ateka, Njeru, & Kibaru, 2004; Gibson, Mwanga, Kasule, Mpembe, & Carey, 1997; Mukasa et al., 2003; Njeru, Bagabe, & Nkezabahizi, 2008; Tairo, Kullaya, & Valkonen, 2004) even despite the poor agronomic practices and conditions conducive to the presence of viral vectors. Viral reversion, which is the absence of viral infection in plants previously infected, was reported by Gibson, Wasswa, and Tufan (2014) in some East African sweetpotato varieties that had been infected with SPFMV. These reverted plants were asymptomatic, and SPFMV was undetectable by the most sensitive test—grafting to *Ipomoea setosa* (Gibson et al., 2014; Moyer, Jackson, & Frison, 1989)—in tested parts of the plant. It is postulated that reversion may explain why SPFMV, which is widely distributed, does not completely devastate sweetpotato crops. Rates of reversion from other, rarer viral infections may be even greater. Reversion may be considered an extreme form of the well-known phenomenon of recovery from viral infection (Gibson & Kreuze, 2015).

Environmental conditions may play a role in a plant’s ability to recover from or reverse viral infection (Ghoshal & Sanfaçon, 2015; Paudel & Sanfaçon, 2018; Qu et al., 2005). In cassava, for example, experiments suggest that higher temperatures increase reversion from infection with African cassava mosaic virus (Gibson & Otim-Nape, 1997). Nutrient status may be another factor that affects rates of reversion (Dordas, 2008; Huber & Graham, 1999), and sweetpotato is often grown in nutrient-depleted soils (Bashasha et al., 1995). In addition, different landraces and cultivars may have a greater or lesser propensity toward viral reversion (Gibson et al., 2014; Mohammed, Ghosh, & Maruthi, 2016).

In this study, we investigated the incidence of viral reversion in response to temperature and soil nutrient enhancements. We used sweetpotato cultivars from East Africa and the United States, after infection or co-infection with viruses of different genera and families.

## 2 MATERIALS AND METHODS

### 2.1 Virus cultures

The East African sweetpotato viruses used were obtained from the National Crops Resources Research Institute in Namulonge, Uganda and Makerere University Agricultural Research Institute in Kabanyolo (MUARIK), central Uganda. MUARIK is 19 km north of Kampala, latitude 0°27′60″N, longitude 32°36′24″E, at an altitude of 1,250–1,320 m above sea level (Yost & Eswaran, 1990). MUARIK receives annual rainfall of about 1,300 mm.

The following viral isolates were partially sequenced to confirm identity (primers are listed in Table 1): SPLCUV (GenBank accession no. FR751068) (Wasswa et al., 2011), SPFMV (East Africa strain; GenBank accession no. FJ795762) (Tugume, Cuéllar, Mukasa, & Valkonen, 2010), SPMMV (GenBank accession no. AJ459319) (Mukasa et al., 2003) and SPCSV (East Africa strain; GenBank accession no. DQ864362) (Aritua, Barg, Gibson, & Vetten, 2008).

**TABLE 1:** Primer sequences used in this study

Virus or gene	Forward and reverse primer names	Primer sequence (5′–3′)	Annealing temperature	Fragment length (bp)	Reference
SPLCUV	SPG3	ACTTCGAGACAGCTATCGTGCC	52°C	1,148	Li, Salih, and Hurtt (2004)
	SPG4	AGCATGGATTCACGCACAGG			
SPCSV	SPCSV-UGF	GACGTTCCGATACGATTGAC	55°C	550	This study
	SPCSV-UGR	TCATCATCAGTGTTGCTGCT			
SPFMV	SPFMV-ILF	CTCCACCACCCACAATAACTG	60°C	810	This study
	SPFMV-ILR	CAGTTGTCGTGTGCCTCTCCG			
SPVC	SPVC-forward	CAAATCAACAGGTTTGCCTTTTTAT	56°C	550	Prakash et al. (2013)
	SPVC-reverse	AGTTCATCGACTTCATTGTAACTTG			
*Actin*	Actin F	GTTATGGTTGGGATGCGACA	58°C	199	Park et al. (2012)
	Actin R	GTGCCTCGGTAAGAAGGACA			
*Cytochrome C oxidase*	Cox F	ACTGGAACAGCCAGAGGAGA	58°C	159	Park et al. (2012)
	Cox R	ATGCAATCTTCCATGGGTTC			

Abbreviations: SPCFV, *Sweet potato chlorotic fleck virus;* SPCLV, *Sweet potato caulimo-like virus;* SPCSV, *Sweet potato chlorotic stunt virus;* SPFMV, *Sweet potato feathery mottle virus;* SPLCUV, *Sweet potato leaf curl Uganda virus;* SPMMV, *Sweet potato mild mottle virus.*

SPVC, family *Potyviridae*, genus *Potyvirus* (GenBank accession no. JX489166; Prakash et al., 2013) was obtained from the Agricultural Research Organization (ARO), The Volcani Center, Israel. Sources of inocula in Uganda were maintained in *I. setosa* and in Israel in coinfected (SPVC + SPCSV) plants of cv. Beauregard.

### 2.2 Nucleic acid extraction and virus identification

For SPLCUV, total nucleic acid extraction was performed from leaves using the CTAB method (Maruthi, Colvin, Seal, Gibson, & Cooper, 2002). For RNA viruses, RNA was extracted from leaves and storage roots using the TRI Reagent protocol following the supplier’s manual (Bio Labs, Jerusalem, Israel). Nucleic acids were quantified using a NanoDrop ND-1000 spectrophotometer (Thermo Scientific; Bargal Analytical Instruments, Airport City, Israel) and evaluated on a 1.5% agarose gel. Complementary DNA (cDNA) was synthesised using Maxima First Strand cDNA Synthesis Kit for RT-qPCR (Thermo Scientific, Tamar, Israel) following the manufacturer’s manual.

Plants in all experiments in Uganda were confirmed infected 1–2 weeks post-inoculation (wpi) using *I. setosa* as an indicator plant and/or by PCR (SPLCUV) or RT-PCR (SPMMV, SPFMV, SPVC, SPCSV); in Israel, infection was confirmed by RT-PCR. For amplification we used a 25-μl PCR master mix of 8.5 μl of water, 12.5 μl of PCR mix (HyLabs Ready Mix [×2], HyLabs, Rehovot, Israel), 1 μl of each primer (10 pmol) and 2 μl of DNA/cDNA (500–1,000 ng/μl). The PCR products were assessed by electrophoresis in a 1.5% agarose gel in 0.5% Tris-acetate buffer, stained with ethidium bromide and viewed under UV light and documented by an OmniDoc gel documentation system (Clever Scientific, Image Care, Uganda).

### 2.3 Sweetpotato cultivars

We used three East African virus-resistant cultivars: New Kawogo, NASPOT 1 (Gasura, Mashingaidze, & Mukasa, 2009) and NASPOT 11 (Mwanga et al., 2011). Two virus-susceptible cultivars from the United States, Resisto and Beauregard, were used as virus-sensitive controls (Gibson et al., 2014).

### 2.4 Reversion from single viral infections

At MUARIK, healthy vine cuttings of 200–250 mm with 3–4 nodes were established in pots with about 1 kg of soil mixture (3:1:1 ratio of black soil: lake sand: cow manure). Plants were grown for 2 weeks in a screenhouse and watered daily.

Plants were side-graft-inoculated with SPLCUV, SPFMV, SPMMV or SPCSV individually using scions of infected *I. setosa* (each scion was ~25 mm). The scions remained on the grafted plants for the experimental period. One mock-inoculated plant per cultivar per virus was included as a control. Plants were tested using *I. setosa* and PCR/RT-PCR at 1 wpi to confirm virus infection. Ten successfully inoculated plants of each cultivar were then evaluated for reversion. Evaluation was done through cutting shoot tips (~50 mm) every 2 weeks for 10 weeks to test for virus infection by side-grafting to *I. setosa* and then testing for the virus presence in *I. setosa* with PCR/RT-PCR. Composite samples of top, middle and basal leaves were used. Removal of shoot tips encouraged lateral shoot growth. Subsequent testing for reversion was performed on these lateral shoots, selecting one shoot every round of testing. Symptom development on *I. setosa* was monitored for 6 weeks and confirmation done with PCR/RT-PCR.

For the evaluation of reversion in roots, vines (~250 mm long with 3–5 nodes) of SPFMV-reverted plants of each of the five cultivars were grown for 16 weeks in a screenhouse in basins of ~25 kg of soil mixture (as above), with five vines per basin. Plants were harvested and three mature storage roots from each basin were sprouted for up to 6 weeks in pots containing ~1 kg of soil mix, with one root per pot. Three leaves (top, middle and basal) were taken weekly from one sprout per bucket (other sprouts were removed) and tested for SPFMV by RT-PCR. Three storage roots from a SPVD-infected plant of cv. NASPOT 1 were sprouted and included as a positive control.

At the ARO, healthy plants of cv. Beauregard were established from vine cuttings with 3–4 nodes (200–250 mm long) in pots containing ~0.5 kg potting mix (Green 90, Evenari Co., Jerusalem, Israel). Plants were grown at 25–27°C and relative humidity of 70–85% in a growth room and watered daily. An SPVD-affected leaf (from a cv. Beauregard plant) was confirmed to be co-infected with SPVC and SPCSV and was used to sap-inoculate (Hull, 2009) seven 1-week-old plants with SPVC. Other plants were mock-inoculated as controls. Sap inoculation transmits only the potyvirus SPVC and not the phloem-limited crinivirus SPCSV.

Plants were tested for SPVC before sap inoculation (Time 0) and thereafter weekly for 4 weeks by RT-PCR. The new leaf above the inoculated leaf was tested for virus at 1 wpi. Subsequently, the third expanded leaf was used. After reversion had occurred, plants were pruned to a stem height of ~200–250 mm leaving only 3–4 leaves. Virus detection was resumed for 2-week and 3-week-old regenerated leaves. Virus detection was also conducted on the roots. Leaf samples of SPVD-affected plants were used as positive controls.

### 2.5 Reversion from SPFMV-SPCSV co-infection

A screen house experiment was set up at MUARIK in a completely randomised design. Week-old plants were infected by side-grafting with a leaf of SPVD (SPFMV + SPCSV)-infected *I. setosa.* The leaf inoculum source was left on the graft-inoculated plants for the experimental period. Shoot tips (~50 mm) were picked from the sweetpotato plants at 3, 5, 7, 9 and 11 wpi and tested for SPFMV and SPCSV infections using *I. setosa.* Cutting shoot tips encouraged growth of lateral shoots. Subsequent testing for reversion was performed using the lateral shoots, selecting one shoot for every round of testing. Observations on *I. setosa* for symptom development were done for 6 weeks. As a control, one plant of each cultivar was mock side-graft-inoculated using healthy *I. setosa.* At the ARO, four SPVD-affected cv. Beauregard plants (co-infected with SPVC + SPCSV) were evaluated for reversion on a weekly basis for 12 weeks using RT-PCR on composite samples of top, middle and basal leaves of the selected shoot.

### 2.6 Reversion from sweetpotato viruses under different temperature regimes

In Uganda, plants of cvs. New Kawogo and Resisto were separately side-graft-inoculated and confirmed as infected with SPLCUV by PCR 1 wpi. Plants from each cultivar were grown at both 20 and 30°C with a 12 hr photoperiod for 4 weeks in a PGI-550H/RH growth chamber (MRC Ltd., Holon, Israel). Healthy controls were included for each temperature regime. The scion inoculum source was left on the graft-inoculated plants for the experimental period. SPLCUV was tested for at the end of experiment using PCR by taking a composite sample of the top, middle and basal leaves.

In a separate experiment, two sets of cv. New Kawogo plants were separately side-graft-inoculated and confirmed as infected with SPFMV using RT-PCR at 1 wpi. One set was grown at 20°C and the other at 30°C with a 12 hr photoperiod for 4 weeks in the growth chamber. Healthy controls were included for each temperature regime. The scion was left on the graft-inoculated plants for the entire experimental period. The presence of SPFMV was tested at the end of experiment by RT-PCR using a composite sample of the top, middle and basal leaves. The numbers of reverted plants in the different categories were tested for significance using chi-square tests.

### 2.7 Reversion from sweetpotato viruses in environments with different temperatures

The field experiments were conducted at MUARIK and at a farm in Kabale District, western Uganda, and were repeated. At MUARIK, growing seasons were September 15 to December 14, 2015 (Season A), and 20 March to June 13, 2016 (Season B). At Kabale, Season A was September 10 to December 9, 2015, and Season B was March 15 to June 13, 2016. During these growing seasons, MUARIK had an average temperature of 22°C and Kabale, more than 2,140 m above sea level, had an average temperature of 19°C. We selected 45 vine cuttings from each of three cultivars (New Kawogo, NASPOT 1 and Resisto) that had been side-graft-inoculated and confirmed as infected with SPFMV using RT-PCR at 1 wpi. Plants were grown in a randomised complete block design with three replicates and planted in single rows on ridges spaced 1 m apart and 0.3 m within the row. Each plot consisted of two rows per cultivar (one SPFMV-infected and one healthy control). Maize was planted between the blocks as a barrier. The presence of SPFMV was tested at 2, 7 and 12 weeks after planting by grafting shoot tips ~50 mm long onto *I. setosa.* Symptom development on *I. setosa* was observed for 6 weeks.

### 2.8 Reversion from SPFMV for plants grown in soil with different enhancements

Four soil treatments (nutrient enhancements) were applied using local cow manure and a commercial fertiliser. The nitrogen (N), phosphorus (P) and potassium (K) fertiliser (NPK) at 17:17:17 (Uganda Crop Care Limited, Juanco SPS Ltd., Nairobi, Kenya) was used according to the manufacturer’s recommendations of 16.67 g per 25 kg soil. The cow manure was well-decomposed, locally prepared manure, obtained from MUARIK, and applied as used by local farmers, ~4 kg per 25 kg soil.

The treatments were as follows: (a) soil only, (b) soil + manure, (c) soil + NPK and (4) soil + half NPK + half manure. The cvs. NASPOT 11, NASPOT 1 and Resisto were used for this experiment, with five SPFMV-infected replicate vines per cultivar. Inoculation was by side-grafting sweetpotato plants with SPFMV-infected scions (~50 mm) of *I. setosa.* Infection was confirmed at 1 wpi by RT-PCR using a composite sample of the top, middle and basal leaves. Five healthy vines of each cultivar were included as controls. Experimental plants were planted in four basins per cultivar per soil treatment, giving a total of 20 vines per cultivar per treatment. Each basin contained 25 kg of field soil, as this was the estimated amount contained in a mound used for growing sweetpotato in the field. The experiment was laid out in a complete block design in a screenhouse at MUARIK. One week after planting the vines, soil amendments were added at the ratios above. Reversion from SPFMV was evaluated seven times over a period of 3 months using RT-PCR, one shoot per test, taking a composite sample of the top, middle and basal leaves.

To evaluate the composition and quality of the soil and amendments before use, a blind analysis, as described by Okalebo, Gathua, and Woomer (2002), was performed for three samples each of the soil, manure and NPK fertiliser to obtain an average composition (Table 2). The analysis was done at the soil laboratory of the College of Agricultural and Environmental Sciences, Makerere University.

**TABLE 2:** Average composition of the amendments used for soil treatments to evaluate their influence on reversion from *Sweet potato feathery mottle virus* (SPFMV)

Amendment	Composition									
	pH	Organic matter (%)	N(%)	P (ppm)	K	Ca	Na	Sand (%)	Clay (%)	Silt (%;
					(cmoles/100 g)					
Soil	6.24	2.28	0.16	7.54	0.22	2.33	0.11	48	38	14
Cow manure	7.32	12.79	0.98	31.18	4.58	14.25	1.24	60.33	20.33	19.33
NPK	8.89	0	7.17	7.18	13.14	1.36	2.97	0	0	0

*Note:* n = 3 per amendment type.

## 3 RESULTS

### 3.1 Reversion from infection with sweetpotato viruses

**FIGURE 1:**
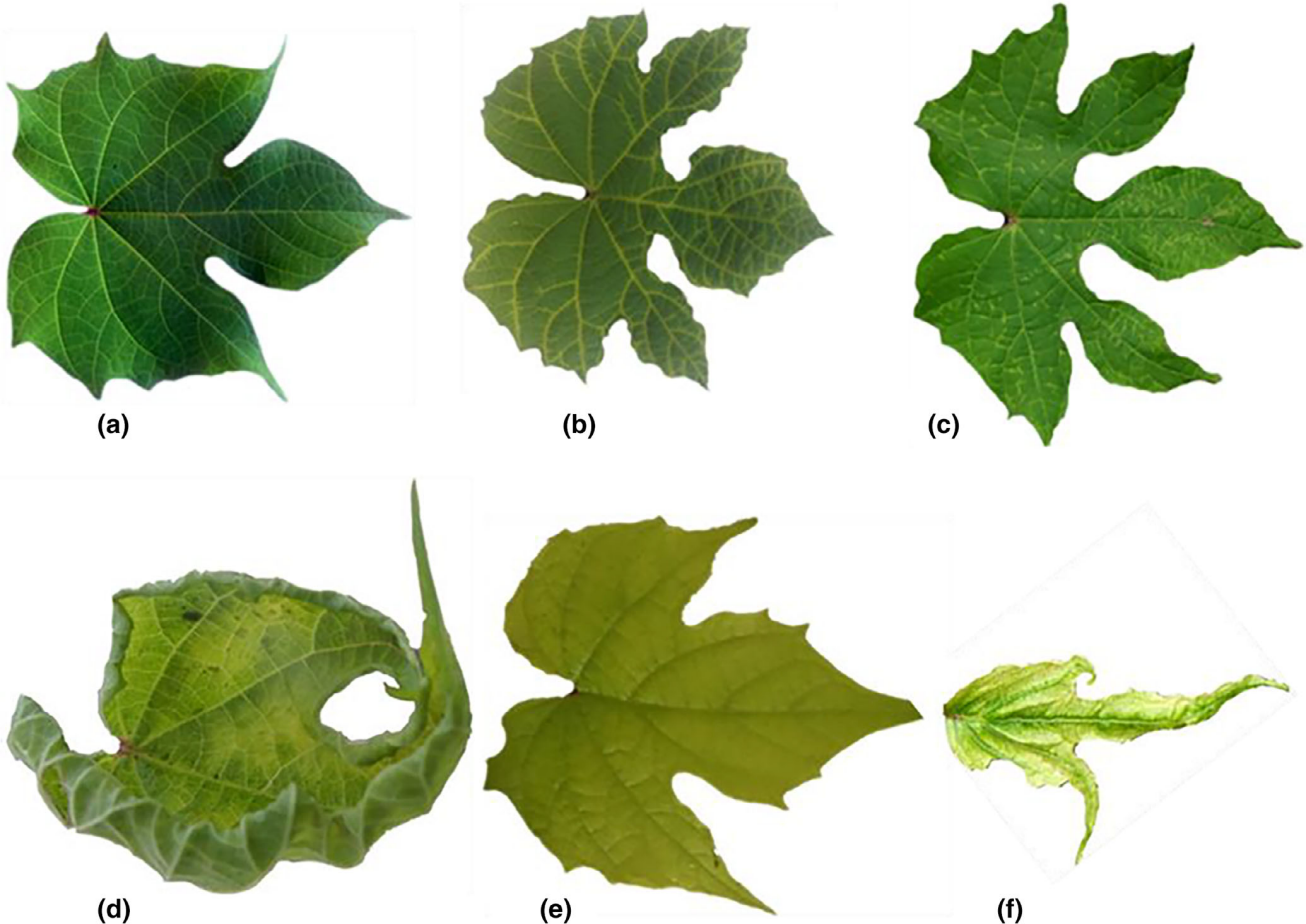
*Ipomoea setosa* leaves showing symptoms commonly induced by sweetpotato viruses. (a) Healthy leaf from an uninfected control plant; (b) vein chlorosis induced by Sweet *potato mild mottle virus* (SPMMV); (c) feathery mottling induced by Sweet *potato feathery mottle virus* (SPFMV); (d) leaf-curling induced by *Sweet potato leaf curl Uganda virus* (SPLCUV); (e) leaf chlorosis induced by *Sweet potato chlorotic stunt virus* (SPCSV); (f) severe mottling, reduced leaf size and leaf distortion due to sweetpotato viral disease (SPVD) induced by dual infection of SPCSV and SPFMV

**FIGURE 2:**
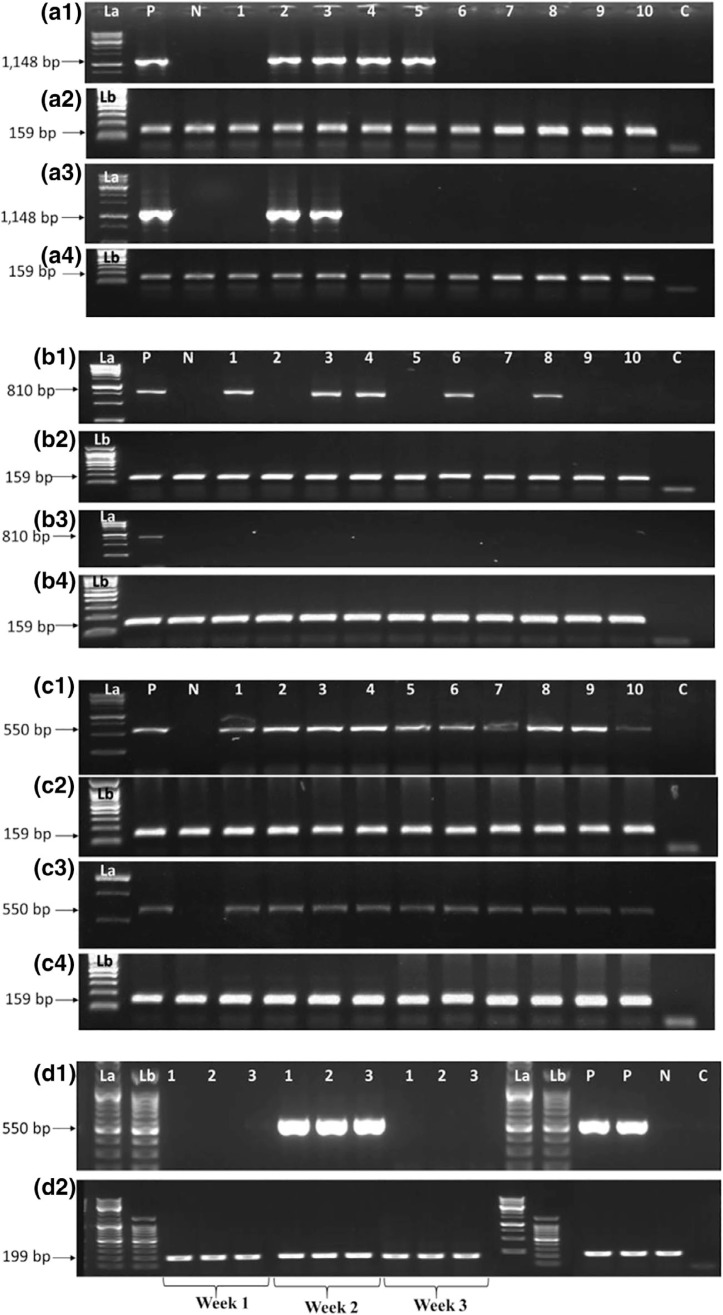
Gels of PCR products showing sweetpotato reversion from infections with different viruses. Each plant was inoculated individually and confirmed to be infected using PCR or RT-PCR prior to the experiment. A plant was considered to have reverted if the virus was completely eliminated from infected plants. This was verified by testing the same plant weekly for 3 weeks. In A1, 6 out of 10 plants of cv. New Kawogo had reverted from SPLCUV by 2 weeks post-inoculation (wpi). In A3, 8 out of 10 plants of cv. New Kawogo had reverted from SPLCUV by 10 wpi. In B1, 5 out of 10 plants of cv. New Kawogo had reverted from SPFMV by 4 wpi. In B3, 10 of 10 plants of cv. New Kawogo had reverted from SPFMV by 10 wpi. In C1, all plants of cv. New Kawogo failed to revert from SPCSV by 4 wpi and similar observations were made in C3 at 10 wpi. D1 is a representative gel for reversion from SPVC in cv. Beauregard following sap inoculation. In Week 1, SPVC was not detected, but was observed in Week 2. In Week 3, the cv. Beauregard plants had reverted. Plates A2, A4, B2, C2 and C4 are the host *cytochrome C oxidase* reference genes. Plates D2 is host *actin* reference gene for the respective samples. Lanes: La = 1-kbp ladder, Lb = 100-bp ladder. Lanes P, N and C are positive, negative and no-template controls, respectively

At MUARIK, reversion from viral infections varied among the virus species and sweetpotato cultivars tested. Single viral infections induced no symptoms in sweetpotato plants but were distinguished by variable symptoms in *I. setosa*, confirmed by PCR/RT-PCR of extracts from *I. setosa* leaves (Figures 1 and 2; Table 1). By 2 wpi, potential reversion from SPMMV and SPLCUV was evident in some plants of all cultivars (Table 3). By 6 wpi, all tested plant parts of East African cvs. New Kawogo and NASPOT 1 (Table 3) appeared to have reverted from SPFMV, although no plant parts of USA cv. Resisto appeared to have reverted from SPFMV. However, the United States cv. Beauregard started to show signs of reversion from SPFMV at 2 wpi, but reverted at a lower rate than the East African cultivars sub-sequently (4 out of 10 at 10 wpi) (Table 3). All plants of cvs. NASPOT 11 and Resisto maintained a reverted state from SPLCUV during Weeks 6–10 (Table 3). All cvs. Beauregard and NASPOT 1 plants had reverted from SPLCUV by 10 wpi; however, only eight of the 10 plants of cv. New Kawogo reverted from SPLCUV by 10 wpi (Table 3 and Figure 2). Also, by 8 weeks, all plants of all cultivars had reverted from SPMMV. Neither East African nor the United States cultivars fully reverted from SPCSV during the entire experimental period (Table 3 and Figure 2). Infection of cv. NASPOT 11 by SPFMV was not observed, and this cultivar was only infrequently infected with SPCSV, SPMMV or SPLCUV (Table 3 and Figure 2). The cv. Resisto reverted effectively from infection by SPLCUV (better than any cultivar except for NASPOT 11), but it reverted poorly (and least effectively of all cultivars) from infection with all other viruses (Table 3).

**TABLE 3:** Reversion from a range of viruses for sweetpotato cultivars tested using *Ipomoea setosa* and PCR/RT-PCR in the screenhouse at MUARIK, Uganda

		No. of plants (out of 18) inoculated	Duration of the experiment (weeks) no. of positives out of a maximum of 10 plants					
			0	2	4	6	8	10
Virus tested	Cultivar							
SPLCUV	New Kawogo	18	10	6	4	3	3	2
	NASPOT 1	12	10	9	3	3	2	0
	NASPOT 11^a^	6	6	3	0	0	0	0
	Resisto	15	10	6	4	0	0	0
	Beauregard	12	10	10	6	3	3	0
SPFMV (East African strain)	New Kawogo	12	10	9	5	0	0	0
	NASPOT 1	18	10	10	6	0	0	0
	NASPOT 11^a^	0	0	0	0	0	0	0
	Resisto	18	10	10	10	10	9	8
	Beauregard	18	10	9	9	6	6	4
SPCSV (East African strain)	New Kawogo	10	10	9	10	10	8	9
	NASPOT 1	18	10	10	10	10	10	10
	NASPOT 11^a^	3	3	3	1	3	3	2
	Resisto	18	10	10	10	10	10	10
	Beauregard	18	10	10	10	10	10	10
SPMMV^b^	New Kawogo	16	10	8	4	0	0	0
	NASPOT 1	18	10	8	6	2	0	0
	NASPOT 11^a^	2	2	0	0	0	0	0
	Resisto	18	10	9	6	1	0	0
	Beauregard	18	10	10	8	3	0	0

*Note:* The *I. setosa* was side-grafted onto the experimental shoots and remained alive for the whole period of the experiment. The starting point for the experiment (0 weeks) was 1–2 weeks after graft inoculation, as this was the first time at which the virus could be detected. For each virus, a maximum of 10 successfully inoculated plants were chosen and followed for reversion. Shoot tips were sampled every 2 weeks for 10 weeks to test for virus infection (and reversion) by grafting to *I. setosa* and further testing with PCR/RT-PCR.

Abbreviations: SPCSV, *Sweet potato chlorotic stunt virus;* SPFMV, *Sweet potato feathery mottle virus;* SPLCUV, *Sweet potato leaf curl Uganda virus;* SPMMV, *Sweet potato mild mottle virus.*

a For each virus, this number of NASPOT 11 plants could be graft-inoculated.

b Detection of SPMMV was performed using *I. setosa.*

Root sprouts from SPVD-affected plants of cv. NASPOT 1 had severe SPVD symptoms and tested positive for SPCSV and SPFMV (data not shown). When roots from all plants of cultivars that had reverted from SPFMV were sprouted, none of the sprouts were SPFMV-infected. All reverted sweetpotato plants had no virus infection symptoms.

At the ARO, no SPVC was observed in healthy cv. Beauregard prior to sap inoculation (at Week 0) (Table 4). The same was true at 1 wpi. At 2 wpi, six of the seven inoculated plants of cv. Beauregard were infected by SPVC, but SPVC was not detected in the same plants at 3 and 4 wpi (Table 4 and Figure 2). When plants were pruned, SPVC was detected in only one plant after regeneration (at 6 wpi) and was not detected at 7 or 8 wpi. Furthermore, SPVC was not detected in any roots of cv. Beauregard at 10 wpi (Table 4 and Figure 2). SPVC was detected in the positive control leaves of SPVD-affected cv. Beauregard plants throughout the experimental period. SPVC was not detected in any mock-inoculated plants.

**TABLE 4:** Summary of viral reversion of *Sweet potato viral C* (SPVC)-infected plants (all cv. Beauregard)

Period post virus inoculation (weeks)									
No. of plants	Initial inoculated shoots					Regenerated plants after pruning			Roots
	0	1	2	3	4	6	7	8	10
Seven SPVC-inoculated	0	0	6	0	0	1	0	0	0
Seven mock-inoculated	0	0	0	0	0	0	0	0	0

*Note:* The experiment was performed in a growth room at the ARO. Confirmation of the lack of viral presence was confirmed by weekly testing using RT-PCR for a period of 10 weeks after sap inoculation. Plants were pruned after 4 weeks, and the new stems were subsequently tested for SPVC infection. Roots were also tested to verify that the whole plant had reverted.

### 3.2 Reversion from co-infection with SPFMV and SPCSV

At MUARIK, five sweetpotato cultivars were graft-inoculated with SPFMV and SPCSV using SPVD-infected *I. setosa* leaves. Sweetpotato plants developed typical SPVD symptoms with cv. NASPOT 11 having milder symptoms by 11 weeks. Virus detection using *I. setosa* revealed differential infection of SPFMV or SPCSV among cultivars over a period of 11 weeks. Within the first 3 weeks, both SPFMV and SPCSV were detected in all plants of cvs. Beauregard, NASPOT 1 and New Kawogo. However, SPFMV and SPCSV were detected in only three and six out of seven plants of cvs. NASPOT 11 and Resisto, respectively. All other plants had single infections of either SPFMV or SPCSV (Table 5). Variable expression of viral symptoms was also observed 5–11 wpi for all cultivars of sweetpotato and *I. setosa* (Table 5). Thus, no cultivar fully reverted from SPVD (Table 5). At the ARO, no reversion from SPVD was observed in cv. Beauregard at 12 wpi (data not shown).

**TABLE 5:** Virus detection in plants co-infected with *Sweet potato feathery mottle virus* (SPFMV) and *Sweet potato chlorotic stunt virus* (SPCSV), which together cause sweet potato viral disease (SPVD)

Cultivar	Week 3	Week 5	Week 7	Week 9	Week 11
Beauregard	7 (2 viruses)	7 (2 viruses)	6 (2 viruses); 1 (SPFMV)	7 (2 viruses)	6 (2 viruses); 1 (SPFMV)
NASPOT 1	7 (2 viruses)	6 (2 viruses); 1 (SPFMV)	6 (2 viruses); 1 (SPFMV)	5 (2 viruses); 1 (SPCSV) 1 (−)	7 (2 viruses)
New
Kawogo	7 (2 viruses)	5 (2 viruses); 2 (SPFMV)	6 (2 viruses); 1 (SPFMV)	7 (2 viruses)	6 (2 viruses); 1 (−)
NASPOT11	3 (2 viruses); 3 (SPFMV); 1 (SPCSV)	7 (2 viruses)	6 (2 viruses); 1 (SPFMV)	3 (2 viruses); 1 (SPFMV); 1 (SPCSV) 2 (−)	4 (2 viruses); 1 (SPFMV); 2 (−)
Resisto	6	(2 viruses);	1 (SPFMV) 6 (2 viruses); 1 (−)	6 (2 viruses); 1 (SPCSV)	6 (2 viruses); 1 (SPFMV)	7 (2 viruses)

*Note:* Seven plants were evaluated for each cultivar for the presence of SPFMV and SPCSV by grafting onto *Ipomoea setosa.* The number of plants where both viruses were detected is given. A “–” indicates that no virus was detected. Shoot tips were sampled from sweetpotato plants at 3, 5, 7, 9 and 11 weeks post-inoculation and tested for SPFMV and SPCSV infections by grafting onto *I. setosa.*

### 3.3 Effect of temperature on reversion

**FIGURE 3:**
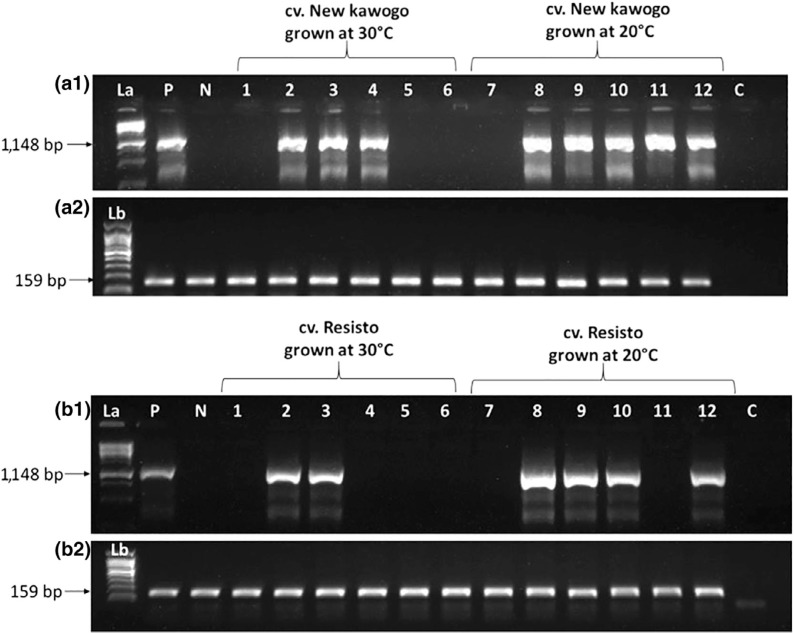
Gels of PCR products showing reversion from *Sweet potato leaf curl Uganda virus* (SPLCUV) when cvs. New Kawogo and Resisto were grown at different temperatures. In A1, 3 out of six plants of cv. New Kawogo had reverted by the fourth week when grown at 30°C. Only one out of six plants of cv. New Kawogo (Lanes 7–12) had reverted by the fourth week when grown at 20°C. In B1, four out of six cv. Resisto plants (Lanes 1–6) had reverted by the fourth week when grown at 30°C and when grown at 20°C, two out of six cv. Resisto plants (Lanes 7–12) had reverted by the fourth week. Plate B2 is host *cytochrome C oxidase* reference gene. Lanes labelled La and Lb are 1-kbp and 100-bp ladders, respectively. Lanes P, N and C are positive, negative and no-template controls respectively

Generally, more plants of cv. Resisto than cv. New Kawogo reverted from SPLCUV at both 20 and 30°C (Table 6 and Figure 3). Additionally, a higher proportion of plants of both cultivars reverted from SPLCUV at 30°C than at 20°C. In the 20°C treatment, only two out of six cv. Resisto plants (33%) reverted from SPLCUV compared with four out of six (67%) that reverted at 30°C. Similarly, only one out of six cv. New Kawogo plants (17%) reverted from SPLCUV at 20°C, compared with three out of six (50%) at 30°C (Table 6 and Figure 3). Similarly, for SPFMV there was substantially more reversion from SPFMV in cv. New Kawogo at 30°C (10 out of 12 plants, 83%) than at 20°C (5 out of 12 plants, 42%) (Table 6 and Figure 3).

**TABLE 6:** Effect of temperature on incidence of reversion from infections of *Sweet potato leaf curl Uganda virus* (SPLCUV) or *Sweet potato feathery mottle virus* (SPFMV)

	No. of plants that reverted from			
	SPLCUV infection	SPFMV infection
Cultivar	20°C	30°C	20°C	30°C
Resisto	2/6	4/6		
New Kawogo	1/6	3/6	5/12	10/12

*Note:* Six plants each of cvs. Resisto and New Kawogo were graft-inoculated with SPLCUV, or six plants of cv. New Kawogo were graft-inoculated with SPFMV, and the infection status of the plants was tested using PCR/RT-PCR after 4 weeks at either 20 or 30°C. Different categories (i.e., for each cultivar at the different temperatures) above were statistically significant at 95% with a chi-squared value equal to 0.079 (df = 1).

### 3.4 Effects of environments with different mean temperatures on reversion

During Season A, the mean field temperatures at MUARIK and Kabale were 22 and 18°C, respectively. Our results indicated more reversion in sweetpotato field plants at MUARIK than at Kabale. At MUARIK, cv. NASPOT 1 reverted most frequently from SPFMV infection (89%), followed by cv. New Kawogo (80%), whereas cv. Resisto seldom reverted (13%) (Table 7). At Kabale, cv. New Kawogo reverted most frequently from SPFMV infection (78%), followed by cv. NASPOT 1 (60%), but cv. Resisto did not revert (0%).

**TABLE 7:** Reversion of sweetpotato cultivars in environments with different mean daytime temperatures

	Season A			Season B		
	MUARIK (average temp. 22°C)			MUARIK (average temp. 21°C)		
	Period after planting (weeks)			Period after planting (weeks)		
Cultivar	2	7	12	2	7	12
Resisto	0	20	13	7	11	13
NASPOT 1	99	87	89	56	51	73
New Kawogo	^a^	^a^	80	87	89	89
	**Kabale (average temp. 18°C)**			**Kabale (average temp. 19.5°C)**		
	**Period after planting (weeks)**			**Period after planting (weeks)**		
Cultivar	2	7	12	2	7	12
Resisto	0	0	0	0	4	4
NASPOT 1	42	42	60	29	38	80
New Kawogo	11	16	78	58	60	80

*Note:* Percentage reversion from *Sweet potato feathery mottle virus* (SPFMV) of graft-infected sweetpotato cultivars (n = 45 plants per treatment) when grown under different temperature regimes for two seasons. Mean daytime temperature for each experiment is given. The presence of SPFMV was tested at 2, 7 and 12 weeks after planting by grafting shoot tips to *Ipomoea setosa*.

a Leaves grazed but the plants regenerated later.

During Season B, average temperatures at MUARIK and Kabale were 21 and 19.5^°^C, respectively. Again, there was generally more reversion at MUARIK than at Kabale. At both MUARIK and Kabale, cv. New Kawogo showed the most reversion with 89 and 80%, respectively. There was more reversion in cv. NASPOT 1 (80%) at Kabale than at MUARIK (73%). At both MUARIK and Kabale, cv. Resisto reverted at the lowest frequency (13 and 4%, respectively) (Table 7).

### 3.5 Reversion of sweetpotato plants grown in soils with varied enhancements

Generally, viral reversion was more prevalent in the treatments in which nutrients were added (Table 8). When soil was amended, more reversion was observed within the first 6 days, especially for cvs. NASPOT 11 and NASPOT 1. The NPK amendment led to the most reversion for all cultivars, followed by the NPK + manure treatment. The addition of amendments, especially NPK or NPK + manure, increased the proportion of cv. Resisto plants that reverted by 3 months compared with the control. All plants of cv. NASPOT 11 and most of cv. NASPOT 1 had reverted from SPFMV by 3 months for all treatments, but fewer cv. Resisto plants had reverted (Table 8).

**TABLE 8:** Effects of soil amendments on reversion of sweetpotato plants from infection with *Sweet potato feathery mottle virus* (SPFMV)

Cultivar	Treatment	Time after soil enhancement						
		3 days	6 days	9 days	12 days	1 month	2 months	3 months
Resisto	Soil only (control)	0	0	0	0	1	5	7
	Soil + NPK	0	1	3	3	8	10	14
	Soil + manure	0	0	0	1	3	8	9
	Soil + NPK + manure	0	2	3	4	9	12	13
NASPOT 1	Soil only (control)	0	1	4	12	18	20	20
	Soil + NPK	1	8	11	16	20	18	20
	Soil + manure	0	5	7	14	20	19	20
	Soil + NPK + manure	1	9	14	15	20	20	18
NASPOT11	Soil only (control)	19	20	19	20	18	20	20
	Soil + NPK	16	20	19	20	20	20	20
	Soil + manure	18	19	20	20	20	20	20
	Soil + NPK + manure	18	20	20	20	20	20	20

*Note:* Values shown indicate the number of reverted plants (out of an initial 20) following graft inoculation with SPFMV, when grown on soil with different soil amendments. The virus was detected using RT-PCR. The start time (0 days) for this experiment was 2 weeks after grafting, when the plants were found to be virus-infected using RT-PCR. Effects of soil amendments on reversion of sweetpotato plants from infection with SPFMV.

## 4 DISCUSSION

Reversion was generally more frequent in East African than the United States sweetpotato cultivars. However, the rate of reversion from viruses of different families varied among the cultivars. For example, cv. New Kawogo reverted more often from SPFMV (potyvirus) than from SPLCUV (begomovirus) infection, whereas cv. Resisto reverted more from SPLCUV than SPFMV (Table 3 and Figure 2). Such variations are consistent with previous field observations in cv. New Kawogo plants by Wasswa et al. (2011). The greater prevalence of SPLCUV in Ugandan sweetpotato fields (Wasswa et al., 2011) is possibly influenced by the low rate of reversion from SPLCUV (a begomovirus) by cv. New Kawogo, despite its ability to revert from SPFMV, as observed here. Clark and Hoy (2006) observed a yield reduction of 26% in begomovirus-infected sweetpotato in the United States, and this virus is perhaps partly responsible for the degeneration in cv. New Kawogo in Uganda. Cv. NASPOT 1 and especially cv. NASPOT 11 consistently reverted from (or were immune to) infection by SPFMV, SPMMV and SPLCUV. We found that cv. NASPOT 11 was very difficult to inoculate with individual viruses of different families (Table 3).

Reversion from SPFMV (Table 3) is consistent with observations by Gibson et al. (2014). Our study is the first to report evidence of reversion from SPMMV and SPLCUV. Plants of both East African and the United States varieties reverted more frequently from SPMMV and SPLCUV than from SPFMV, and this could explain the comparatively low field prevalence of SPMMV and SPLCUV observed in East Africa (Aritua, Legg, Smita, & Gibson, 1999) and the United States (Clark et al., 2012). Furthermore, shoots that regenerated from pruned reverted plants or root sprouts from reverted plants tested negative for the viruses, indicating complete reversion. This is the first report to show complete reversion from viruses in sweetpotato plants from the United States (Tables 3 and 4). Reversion was very effective in cv. Beauregard following sap inoculation (Table 4), which was much less aggressive than graft inoculation (Table 3). Reversion may be considered to be an extreme form of recovery (Basu et al., 2018; Gibson & Kreuze, 2015; Paudel & Sanfaçon, 2018).

None of the five tested cultivars (including cv. NASPOT 11) effectively reverted from infections involving SPCSV, including the SPFMV + SPCSV co-infection (Tables 3 and 5). The incomplete reversion from SPFMV + SPCSV combination was also observed by Mwanga, Yencho, Gibson, and Moyer (2013). In their study, some severely SPVD-affected genotypes developed localised symptoms from which they recovered. These recovered plants could only subsequently produce whole branches or individual shoots that had apparently reverted, being free of detectable virus when assayed by ELISA or grafted onto *I. setosa*.

Despite the failure of plants to effectively revert from SPCSV (and thus SPVD), this virus is rarer than SPFMV in the field, possibly for two reasons: (a) symptoms of SPCSV infection are more evident than for the other viruses, so farmers select against taking cuttings from affected plants and (b) greater transmission of SPFMV. SPFMV can spread very rapidly in the field, in Israel reaching 51–84% infection in a single growing cycle for plants near infected plots (Milgram, Cohen, & Loebenstein, 1996). In a field in Brazil, 80% of experimental plants were SPFMV-infected by 10 weeks (Pozzer, Dusi, Silva, & Kitajima, 1994). In the United States, 100% infection by SPFMV can occur within 5–10 weeks (Bryan, 2002). Failure of plants to revert from SPCSV may be due to the presence of the *p22* and *RNase 3* silencing suppressor genes in the East African strain of SPCSV. These genes suppress the gene-silencing mechanism of the plant (Cuellar, Tairo, Kreuze, & Valkonen, 2008), and thus probably compromise plant defences to the extent that reversion cannot effectively occur and other viruses can easily infect (Kreuze, Savenkov, Cuellar, Li, & Valkonen, 2005), causing SPVD. This effect of SPCSV infection also may explain why plants failed to fully revert from a SPFMV + SPCSV co-infection (SPVD).

Under laboratory conditions, at an elevated temperature of 30°C, the reversion of the United States cv. Resisto from SPLCUV was greatly enhanced (Table 6 and Figure 3), and cv. New Kawogo also reverted effectively from two different virus families (Table 6 and Figure 3) at 30°C. Additionally, in different environments with temperature differences, field plants more frequently reverted when grown at higher (21–22°C) compared with lower temperatures (18–19.5^°^C) (Table 7), although we acknowledge that numerous other factors may have been at play in these fields. In the field experiments, reversion occurred more strongly for East African compared with the United States cultivars. These results together with the temperature-controlled experiments suggest that temperature may directly affect the reversion process. Similar results were observed by Rossel and Thottappilly (1985) in tropical root crops, Bertschinger, Keller, and Gessler (1995) in potato, Gibson and Otim-Nape (1997) in cassava and Aritua, Alicai, Adipala, Carey, and Gibson (1998) in sweetpotato cvs. New Kawogo and Tanzania. These studies indicated that better rates of viral reversion are likely due to increased plant defence RNA interference mechanisms at high compared to low temperatures (Chellappan, Vanitharani, Ogbe, & Fauquet, 2005).

More plants reverted from SPFMV when grown in soil fertilised with NPK or with a NPK–manure combination, but not in manure alone, compared with unamended soil (Table 8). It is probable that this occurred because the soil amendments (nutritional supplements) enhanced plant vigour and growth, as has been observed for radish (Sarker, Kashem, & Osman, 2012). Well-fertilised plants generally have more resources to fight infection. The use of soil nutrients to manage plant diseases caused by fungi and bacteria has been noted previously (Dordas, 2008); phosphorus specifically has been reported to enhance plant defences against viral disease (Huber & Graham, 1999). However, fertilisation is not a panacea for viral infection; Bhaduri, Rakshit, and Chakraborty (2014) found that in the presence of an external N supply (such as from NPK), visible symptoms (e.g., of SPFMV) are dependent upon competition for N between the virus and host cells.

The reversion from viral infection in sweetpotato observed here was virus-specific and affected by environmental factors. The contribution of genotype to the predisposition to revert was evident for sweetpotato cultivars from both East Africa and the United States, suggesting a generality to this phenomenon, which requires further study. Research on the mechanisms of reversion and the environmental conditions that improve reversion rates could provide additional methods to control sweetpotato viruses. Further investigation of the incidence of reversion in different sweetpotato cultivars could improve breeding and production of virus-free planting material without the use of expensive in vitro virus-elimination techniques.

## References

[cit0001] Adikini S., Mukasa S. B., Mwanga R. O., & Gibson R. W. (2015). Sweet potato cultivar degeneration rate under high and low sweet potato virus disease pressure zones in Uganda. *Canadian Journal of Plant Pathology*, 37, 136–147.

[cit0002] Andrade M., Barker I., Cole D., Dapaah H., Elliott H., Fuentes S., … Thiele G. (2009) Unleashing the potential of sweetpotato in sub-Saharan Africa: Current challenges and way forward. International Potato Center (CIP), Lima, Peru Retrieved from https://issuu.com/internationalpotatocenter/docs/004718

[cit0003] Aritua V., Alicai T., Adipala E., Carey E. E., & Gibson R. W. (1998). Aspects of resistance to sweet potato virus disease in sweet potato. *Annals of Applied Biology*, 132, 387–398.

[cit0004] Aritua V., Barg E., Gibson R. W., & Vetten J. H. (2008). Further evidence for limited genetic diversity among east African isolate of *Sweet potato chlorotic stunt virus*. *Journal of Phytopathology*, 156, 181–189.

[cit0005] Aritua V., Bua B., Barg E., Vetten H. J., Adipala E., & Gibson R. W. (2007). Incidence of five viruses infecting sweet potatoes in Uganda; the first evidence of sweet potato caulimo-like virus in Africa. *Plant Pathology Journal*, 56, 324–331.

[cit0006] Aritua V., Legg J. P., Smita N. E. J. M., & Gibson R. W. (1999). Effect of local inoculum on the spread of sweet potato virus disease: Limited infection of susceptible cultivars following widespread cultivation of a resistant sweetpotato cultivar. *Plant Pathology Journal*, 48, 655–661.

[cit0007] Ateka E. M., Njeru R. W., & Kibaru A. G. (2004). Identification and distribution of viruses infecting sweet potato in Kenya. *Annals of Applied Biology*, 144, 371–379.

[cit0008] Bashasha B., Mwanga R. O. M., Ocitti p’Obwoya C., & Ewell P. T. (1995) *Sweetpotato in farming and food systems of Uganda. A farm survey report*. Sub-Saharan African Region, Nairobi, Kenya/National Agricultural Research Organization, Kampala, Uganda Retrieved from http://citeseerx.ist.psu.edu/viewdoc/download?doi=10.1.1.534.7117&rep=rep1&type=pdf

[cit0009] Basu S., Kumar K. N., Kumar S. A., Pankaj S. P., Vinoth K. R., & Chakraborty S. (2018). Dynamics of a geminivirus-encoded pre-coat protein and host RNA-dependent RNA polymerase 1 in regulating symptom recovery in tobacco. *Journal of Experimental Botany*, 69, 2085–2102. 29432546 10.1093/jxb/ery043 PMC6019014

[cit0010] Bertschinger L., Keller E. R., & Gessler C. (1995). Characterization of the virus x temperature interaction in secondarily infected potato plants using EPIVIT. *Phytopathology*, 85, 815–819.

[cit0011] Bhaduri D., Rakshit R., & Chakraborty K. (2014). Primary and secondary nutrients – A boon to defense system against plant diseases. *International Journal of Stress Management*, 5, 461–466.

[cit0012] Bryan A. D. (2002) Impact of Sweet potato feathery mottle virus and micropropagation on yield, root quality, and virus incidence in commercial sweetpotato production systems. (Master’s thesis). University of North Carolina, Chapel Hill, NC.

[cit0013] Chellappan P., Vanitharani R., Ogbe F., & Fauquet C. M. (2005). Effect of temperature on geminivirus-induced RNA silencing in plants. *Plant Physiology*, 138(4), 1828–1841. 16040661 10.1104/pp.105.066563 PMC1183375

[cit0014] Clark C. A., Davis J. A., Abad J. A., Cuellar W. J., Fuentes S., Kreuze J. F., … Valkonen J. P. T. (2012). Sweetpotato viruses: 15 years of progress on understanding and managing complex diseases. *Plant Disease*, 96, 168–185. 30731810 10.1094/PDIS-07-11-0550

[cit0015] Clark C. A., & Hoy M. W. (2006). Effects of common viruses on yield and quality of Beauregard sweetpotato in Louisiana. *Plant Disease*, 90, 83–88. 30786480 10.1094/PD-90-0083

[cit0016] Cuellar W. J., Tairo F., Kreuze J. F., & Valkonen J. P. T. (2008). Analysis of gene content in *Sweet potato chlorotic stunt virus* RNA1 reveals the presence of p22 RNA silencing suppressor in only few isolates: Implications to viral evolution and synergism. *Journal of General Virology*, 89, 573–582. 18198389 10.1099/vir.0.83471-0

[cit0017] Dordas C. (2008). Role of nutrients in controlling plant diseases in sustainable agriculture. A review. *Agronomy for Sustainable Development*, 28, 33–46.

[cit0018] Food and Agriculture Organization (2016) Production quantity of primary crops, country statistics for eastern Africa. Retrieved from http://www.fao.org/faostat/en/#data/QC

[cit0019] Gasura E., Mashingaidze A. B., & Mukasa S. B. (2009). Occurrence, prevalence and implications of sweetpotato recovery from sweet potato virus disease in Uganda. *African Crop Science Journal*, 9, 601–608.

[cit0020] Ghoshal B., & Sanfaçon H. (2015). Symptom recovery in virus-infected plants: Revisiting the role of RNA silencing mechanisms. *Virology*, 479, 167–179. 25677651 10.1016/j.virol.2015.01.008

[cit0021] Gibson R. W., & Kreuze J. F. (2015). Degeneration in sweetpotato due to viruses, virus-cleaned planting material and reversion: A review. *Plant Pathology*, 64, 1–15.

[cit0022] Gibson R. W., Mpembe J., Alicai T., Carey E. E., Mwanga R. O. M., Seal S. E., & Vetten H. J. (1998). Symptoms, etiology and serological analysis of sweetpotato virus diseases in Uganda. *Plant Pathology Journal*, 47, 95–102.

[cit0023] Gibson R. W., Mwanga R. O. M., Kasule S., Mpembe I., & Carey E. E. (1997). Apparent absence of viruses in most symptomless field-grown sweetpotato in Uganda. *Annals of Applied Biology*, 130, 481–490.

[cit0024] Gibson R. W., & Otim-Nape G. W. (1997). Factors determining recovery and reversion in mosaic-affected African cassava mosaic resistant cassava. *Annals of Applied Biology*, 131, 259–271.

[cit0025] Gibson R. W., Wasswa P., & Tufan H. A. (2014). The ability of cultivars of sweetpotato in East Africa to revert from *Sweet potato feathery mottle virus* infection. *Virus Research*, 186, 130–134. 24361352 10.1016/j.virusres.2013.12.006

[cit0026] Huber D. M., & Graham R. D. (1999). The role of nutrition in crop resistance and tolerance to disease. In Rengel Z. (Ed.), *Mineral nutrition of crops fundamental mechanisms and implications* (pp. 205–226). New York: Food Product Press.

[cit0027] Hull R. (2009). Mechanical inoculation of plant viruses. *Current Protocols in Microbiology*, 16, 11–14. 10.1002/9780471729259.mc16b06s13 19412912

[cit0028] Karyeija R. F., Gibson R. W., & Valkonen J. P. T. (1998). Resistance to sweet potato virus disease (SPVD) in wild east African *Ipomoea*. *Annals of Applied Biology*, 133, 39–44.

[cit0029] Kreuze J. F., Savenkov E. I., Cuellar W., Li X., & Valkonen J. P. T. (2005). Viral class 1 RNase III involved in suppression of RNA silencing. *Journal of Virology*, 79, 7227–7238. 15890961 10.1128/JVI.79.11.7227-7238.2005 PMC1112141

[cit0030] Li R., Salih S., & Hurtt S. (2004). Detection of geminiviruses in sweetpotato by polymerase chain reaction. *Plant Disease*, 88, 1347–1351. 30795196 10.1094/PDIS.2004.88.12.1347

[cit0031] Maruthi M. N., Colvin J., Seal S., Gibson G., & Cooper J. (2002). Coadaptation between cassava mosaic geminiviruses and their local vector populations. *Virus Research*, 86, 71–85. 12076831 10.1016/s0168-1702(02)00051-5

[cit0032] Milgram M., Cohen J., & Loebenstein G. (1996). Effects of sweet potato feathery mottle virus and sweet potato sunken vein virus on sweet potato yields and rates of reinfection of virus-free planting material in Israel. *Phytoparasitica*, 24, 189–193.

[cit0033] Mohammed I. U., Ghosh S., & Maruthi M. N. (2016). Host and virus effects on reversion in cassava affected by cassava brown streak disease. *Plant Pathology*, 65, 593–600.

[cit0034] Mukasa S. B., Rubaihayo P. R., & Valkonen J. P. T. (2003). Incidence of viruses and virus like diseases of sweetpotato in Uganda. *Plant Disease*, 87, 329–335. 30831824 10.1094/PDIS.2003.87.4.329

[cit0035] Mwanga R. O. M., Niringiye C., Alajo A., Kigozi B., Namakula J., Mpembe I., … Yencho G. C. (2011). ‘NASPOT 11’, a sweetpotato cultivar bred by a participatory plant-breeding approach in Uganda. *HortScience*, 46, 317–321.

[cit0036] Mwanga R. O. M., Yencho G. C., Gibson R. W., & Moyer J. W. (2013). Methodology for inoculating sweetpotato virus disease: Discovery of tip dieback, and plant recovery and reversion in different clones. *Plant Disease*, 97, 30–36. 30722256 10.1094/PDIS-12-11-1072-RE

[cit0037] Moyer J. W., Jackson G. V. H., & Frison E. A. (1989). *FAO/IBPGR technical guidelines for the safe movement of sweetpotato germplasm*. Rome, Italy: FAO/International Board.

[cit0038] Njeru R. W., Bagabe M. C., & Nkezabahizi D. (2008). Viruses infecting sweet potato in Rwanda: Occurrence and distribution. *Annals of Applied Biology*, 153, 215–221.

[cit0039] Okalebo R. J., Gathua K. W., & Woomer P. L. (2002). *Laboratory methods of soil and plant analysis: A working manual* (2nd ed.). Nairobi, Kenya: Sacred African Publishers.

[cit0040] Park S. C., Kim Y. H., Ji C. Y., Park S., Jeong J. C., & Lee H. S. (2012). Stable internal reference genes for the normalization of real-time PCR in different sweetpotato cultivars subjected to abiotic stress conditions. *PLoS One*, 7, e51502. 23251557 10.1371/journal.pone.0051502 PMC3520839

[cit0041] Paudel D. B., & Sanfaçon H. (2018). Exploring the diversity of mechanisms associated with plant tolerance to virus infection. *Frontiers in Plant Science*, 9, 1575. 30450108 10.3389/fpls.2018.01575 PMC6224807

[cit0042] Pozzer L., Dusi A. N., Silva J. B. C., & Kitajima E. W. (1994). The rate of reinfection of virus-free sweetpotato plants by *Sweet potato feathery mottle virus*, under field conditions. *Fitopatologia Brasileira*, 19, 231–234 (Portuguese, with English abstr.).

[cit0043] Prakash S., Tam Y., Zeidan M., Abu-Ras A., & Gaba V. (2013). First report of *Sweet potato virus C* infecting sweet potato in Israel. *New Disease Report*, 28, 4.

[cit0044] Qu F., Ye X., Hou G., Sato S., Clemente T. E., & Morris T. J. (2005). The *Nicotiana benthamiana* homolog of SDE1/SGS2/RDR6 functions in a temperature-dependent manner to defend both differentiated tissues and shoot apices against viral invasion. *Journal of Virology*, 79, 15209–15217. 16306592 10.1128/JVI.79.24.15209-15217.2005 PMC1316014

[cit0045] Rachkara P., Phillips D. P., Kalule S. W., & Gibson R. W. (2017). Innovative and beneficial informal sweetpotato seed private enterprise in northern Uganda. *Food Security*, 9, 595–610. 10.1007/s12571-017-0680-4 PMC7473088 32968464

[cit0046] Rossel H. W., & Thottappilly G. (1985). *Virus diseases of important food crops in tropical Africa*. Ibadan, Nigeria: International Institute of Tropical Agriculture.

[cit0047] Sarker A., Kashem M. A., & Osman K. T. (2012). Comparative effect of city finished compost and NPK fertilizer on growth and availability of phosphorus to radish (*Raphanus sativus* L.). *Open Journal of Soil Science*, 2, 146–154.

[cit0048] Tairo F., Kullaya A., & Valkonen J. P. T. (2004). Incidence of viruses infecting sweetpotato in Tanzania. *Plant Disease*, 88, 916–920. 30812241 10.1094/PDIS.2004.88.9.916

[cit0049] Tairo F., Mukasa S. B., Jones R. A. C., Kullaya A., Rubaihayo P. R., & Valkonen J. P. T. (2005). Unravelling the genetic diversity of the three main viruses involved in sweetpotato virus disease (SPVD) and its practical implications. *Molecular Plant Pathology*, 6, 199–211. 20565651 10.1111/j.1364-3703.2005.00267.x

[cit0050] Tugume A. K., Cuéllar W. J., Mukasa S. B., & Valkonen J. P. T. (2010). Molecular genetic analysis of virus isolates from wild and cultivated plants demonstrates that East Africa is a hotspot for the evolution and diversification of *Sweet potato feathery mottle virus*. *Molecular Ecology*, 19, 3139–3156. 20609081 10.1111/j.1365-294X.2010.04682.x

[cit0051] Wasswa P., Otto B., Maruthi M. N., Mukasa S. B., Monger W., & Gibson R. W. (2011). First identification of a sweet potato begomovirus (sweepovirus) in Uganda: Characterization, detection and distribution. *Plant Pathology*, 60, 1030–1039.

[cit0052] Yost D., & Eswaran H. (1990). *Major land resource areas of Uganda*. Washington, DC: World Soil Resources, Soil Conservation Service-USDA.

